# Cancer worry frequency vs. intensity and self-reported colorectal cancer screening uptake: A population-based study

**DOI:** 10.1177/0969141319842331

**Published:** 2019-05-01

**Authors:** Charlotte Vrinten, Sandro Stoffel, Rachael H Dodd, Jo Waller, Yoryos Lyratzopoulos, Christian von Wagner

**Affiliations:** Department of Behavioural Science and Health, University College London, London, UK

**Keywords:** Cancer, fear, worry, anxiety, colorectal cancer, screening, faecal occult blood test

## Abstract

**Objectives:**

Many studies of cancer worry use items measuring frequency or intensity. Little is known about how each of these relate to cancer screening uptake. This study compared the association between worry frequency vs. intensity and colorectal cancer screening intention/uptake.

**Methods:**

Across four surveys (2014–2016), we collected data from 2878 screening-eligible men and women (aged 60–70) in England. Measures included single-items assessing cancer worry frequency and intensity, and a derived combination of both. We also assessed self-reported past faecal occult blood testing uptake (ever vs. never), intention to participate when next invited (yes vs. no), and demographics. Using logistic regression, we compared a model containing sociodemographic characteristics (Model 1), with four models adding cancer worry frequency (Model 2), intensity (Model 3), both (Model 4), or the combined measure (Model 5).

**Results:**

A model with cancer worry intensity and demographics (Model 3) explained significantly more variance in uptake and intention (*R*^2^ = 0.068 and 0.062, respectively) than demographics alone (Model 1: *R*^2^ = 0.058 and 0.042; *p* < 0.001), or a model with demographics and cancer worry frequency (Model 2: *R*^2^ = 0.059 and 0.052; *p* < 0.001). The model was also equally as effective as models including both the frequency and intensity items (Model 4: *R*^2^ = 0.070 n.s. and 0.062 n.s.), or using the derived combination of both (Model 5: *R*^2^ = 0.063 n.s. and 0.053 n.s.).

**Conclusion:**

A single item measure of cancer worry intensity appeared to be most parsimonious for explaining variance in colorectal cancer screening intention and uptake.

## Introduction

Colorectal cancer (CRC), the fourth most common cancer worldwide, accounts for 8.5% of all cancer mortality.^1^ In the UK, nearly 35,000 new cases are diagnosed annually, and CRC accounts for 10% of all cancer deaths.^2^ Population-based screening using the faecal occult blood test (FOBt) can contribute to reducing the relative risk of CRC mortality by up to 25% in those completing at least one round of screening.^3^ Since 2006, the Bowel Cancer Screening Programme (BCSP) in England has invited men and women aged 60–69 to undertake free, home-based biennial FOBt screening for colorectal cancer (this programme has since been extended to age 74). Uptake is sub-optimal (56% in 2015–16), with known socio-demographic variation (particularly lower uptake among ethnic minorities and individuals living in poorer areas^4–7^), and effects of practical and psychological factors.^6^ One such psychological factor is cancer worry, a negative emotional reaction to the threat of cancer,^8^ shown to be higher in women, those who are younger, and those from lower socioeconomic or ethnic minority backgrounds.^9,10^

The effect of cancer worry on CRC screening uptake is uncertain. Some studies have found a positive association,^11–13^ others a negative association,^14,15^ some both positive and negative associations when considering different facets of cancer worry,^5,16–18^ and others finding no effect.^19–24^ Comparison between studies is hindered by inconsistencies in how cancer worry is operationalized.^8,25,26^ Although studies should ideally include comprehensive measures of cancer worry, few have the capacity to include detailed measures. Due to space restrictions, most large population-based studies can only measure general worry about cancer, without further specification of the object of the worry. When measuring general cancer worry, some studies operationalize it in terms of the *frequency* of fearful thoughts about cancer, while others measure the *intensity* of fearful thoughts about cancer. In our view, cancer worry frequency and intensity are two different, yet correlated, dimensions of cancer worry, on which people may score differently. For example, someone may feel very anxious when they think about cancer (high intensity of cancer worry), and therefore try to avoid thinking about it (low frequency), suggestive of an avoidant coping style for cancer risk. Indeed, a study of underutilizers of breast cancer screening^27^ found that women who were not planning to have a mammogram tended to be “not at all” worried about breast cancer, yet also tended to agree that they were “so fearful of cancer that you don’t want to know if you have cancer or not”.

The idea of cancer worry as a function of both frequency and intensity of cancer worry is not new. Scales of fear of cancer recurrence often include both,^28^ and clinical anxiety disorders are also characterized in terms of being excessive (intensity) or persistent (frequency, duration) compared with normative fear or anxiety (leading to clinically important distress or impairment in functioning, unlike cancer worry in the general population as here described^29^). However, most population-based surveys of cancer worry include only one of these dimensions, so it is difficult to establish whether different associations with behavioural outcomes found across studies are due to differences in measurement or to real differences in the behavioural correlates of cancer worry frequency versus intensity.

To our knowledge, only three large population-based studies have simultaneously included items that can be considered as measures of cancer worry frequency and cancer worry intensity to examine associations with uptake of CRC screening,^16–18^ and thus allow comparisons between these two measures and their association with cancer screening behaviour. Using data from the US Health Information National Trends Survey (HINTS), McQueen et al.^16^ did not find an association between *cancer worry frequency* (i.e. “How often do you worry about getting colon cancer?”) and uptake of FOBt or endoscopic screening. However, *being afraid of finding cancer*, if tested (i.e. agreeing to the statement “You are afraid of finding colon cancer if you were checked”), was associated with a higher likelihood of endoscopic screening in women, and also seemed to be associated with higher uptake of FOBt, although this did not reach statistical significance. Wong et al.^18^ used similar measures of *cancer worry frequency* (i.e. (dis)agreement with the statement “I never worry about getting CRC”) and *fear of finding cancer* (i.e. “I am afraid of finding out if I have CRC”) in a population-representative survey in Singapore to examine associations with CRC screening uptake (either FOBT or endoscopic screening, although FOBt was reportedly used most often as test modality). Their findings showed that more frequent worry about getting CRC was associated with higher uptake of screening, while fear of finding out about CRC was associated with lower uptake.^18^ Finally, in a prospective study of flexible sigmoidoscopy (FS) screening in the UK, Vrinten et al.^17^ found that *worrying a lot about cancer* (i.e. agreement with the statement “I worry a lot about cancer”) was associated with a higher likelihood of intending to attend screening, but did not predict actual attendance, while *feeling uncomfortable when thinking about cancer* (i.e. “It makes me uncomfortable to think about cancer”) was associated with a lower likelihood of attending FS screening.^17^ These mixed findings suggest that there may be differences between cancer worry frequency and intensity measures and their relationship with screening intentions and uptake, although the study samples were very different and no firm conclusions can therefore be drawn from these previous findings. In addition, these studies only reported the behavioural associations for items separately, and did not examine whether the behavioural associations of cancer worry are even stronger for a combined index of frequency and intensity of cancer worry, as may be hypothesized based on the characterization of clinically relevant anxiety as described above.

To better understand how cancer worry can best be measured in population-based studies that seek to examine the association of cancer worry with cancer-related behavioural outcomes, this study compared measures of cancer worry frequency, cancer worry intensity and a combination of both, and their association with intention and uptake of the FOBT in a population-based sample of screening-eligible English adults aged 60–70.

## Methods

### Design

We combined data from all four waves of the Attitudes, Behaviour and Cancer UK Survey (Attitudes, Behaviour and Cancer UK Survey [ABACUS]; 2014–2016), which were collected by TNS Research International as part of their weekly omnibus survey using face-to-face, computer-assisted personal interviews. The TNS omnibus survey defines sample points using 2001 Census small-area statistics and the Postcode Address File (stratified by social grade and Government Office Region), which are used for random location sampling. To ensure a population-representative sample, quotas are set for age, sex, children in the home, and working status, and weights are provided by the survey company. An adjustment weight was assigned to each case during analysis, so that respondents with characteristics (age, sex, social grade, region) who were underrepresented (relative to the national population) were given a higher weight (>1) than those who were relatively overrepresented (weight <1). The ABACUS survey is a series of four population-based surveys designed to assess attitudes to cancer and cancer screening in England from 2014 to 2016. The first (2014; *N* = 1675) focused mainly on colorectal cancer screening using FOBt and included men and women aged 58–70. The second (2015; *N* = 1464) focused on colorectal cancer screening using FOBt and FS and included a wider age range (men and women aged 50–70), while the third (*N* = 2111) and fourth surveys (*N* = 2048; both collected in 2016) focused on various beliefs and attitudes about cancer in the general population, and included men and women aged 18–70. The upper age limit for the survey was chosen because this is the age at which people tend to stop being invited to cancer screening in England; the extension of the colorectal cancer screening programme to age 74 had not been fully rolled out across England at the time of the surveys. Several other papers have been published from these datasets, including some that examine different objects of cancer worry (e.g. fear of death) in relation to cancer screening (ABACUS wave 3),^10,30^ but this is the first time that we have combined all four datasets to obtain a large enough sample to examine the role of cancer worry frequency and intensity on colorectal cancer screening uptake. All respondents resided in England at the time of the interview.

### Participants

The sample for these analyses consisted of 2878 men and women aged 60–70. We excluded participants who were outside this age range and those who had a previous diagnosis of cancer. All participants consented at the start of the interview.

### Measures

Sociodemographics, including the respondents’ age, gender, ethnicity, marital status, and social grade were recorded using simple questions. Ethnicity was measured using 16 categories from the UK Census,^31^ but were dichotomized into White vs. non-White (including mixed ethnic backgrounds) due to small numbers in the ethnic minority groups. Marital status was recorded as “married or living as married,” “single,” and “widowed, separated, or divorced.” Social grade was used as an indicator of socioeconomic status and was recorded using the categories from the National Readership Survey.^32^ These are based on the occupation of the chief wage earner in each household. The categories were: A (higher managerial, administrative, or professional), B (intermediate managerial, administrative, or professional), C1 (supervisory, clerical or junior managerial, administrative, or professional), C2 (skilled manual), D (semiskilled or unskilled manual), or E (state pensioners, casual/lowest grade workers, or unemployed with state benefits only). Grades A and B were combined to create more equal sized groups, as were groups D and E.

Cancer worry was assessed with two items, one assessing *cancer worry frequency* and one assessing *cancer worry intensity.* These questions were the same across all four surveys. Cancer worry frequency was measured with the item: ‘How often do you worry about your chance of getting cancer yourself?’, with response options ‘never’, ‘occasionally’, ‘sometimes’, ‘often’, and ‘very often’, which was adapted from the US HINTS.^33^ Cancer worry intensity was measured with the item: ‘How anxious do you feel when you think about cancer?’, with response options ‘not at all’, ‘slightly’, ‘quite a bit’, and ‘extremely’. The two items were significantly correlated (r_s_[2632] = 0.573, *p* < 0.001). It should be noted that these items are about cancer in general, and are not specific to colorectal cancer. In addition, we combined these two items to create a conceptual categorization of cancer worry. In accordance with accepted definitions of clinically relevant anxiety, which differentiates anxiety disorders from developmentally normative fear as being “excessive or persisting beyond developmentally appropriate periods”^29^ (i.e. as a function of intensity or frequency/duration of the anxiety), we conceptualized high cancer worry as worry that was very high in intensity, or very frequent. Consistent with some of our previous studies,^34,35^ we categorized this combined measure in three levels: “no cancer worry” (those who “never” worried and were “not at all” anxious), moderate cancer worry (those who worried “occasionally” or “sometimes”, or who were “slightly anxious”), and high cancer worry (those who worried “often” or “very often”, or who were “quite a bit” or “extremely” anxious). We have favoured a conceptual categorization in three categories (to allow for exploration of curvilinear effects) rather than combining the two items by adding or averaging the scores, because scores in the middle of the range for sum or average scores may be difficult to interpret. For example, they could result from scores in the middle of the range for both items, or from scores at the top end of the range for one item and the bottom end of the range for the other item. Conceptually, these two scenarios are very different from each other, and we have therefore favoured a conceptual classification, whereby high scores on either (or both) item(s) would always result in being classified as being ‘high’ in cancer worry, while only scoring the lowest score on both items (‘not at all anxious’ and ‘never worried’) results in being classified as having ‘no’ cancer worry.

Intentions to do the FOB test in the future were assessed in all surveys using variations of the same question: “Will you do the stool test when you are next sent one/the next time you are sent a kit/the next time you are invited?” Response options were the same for all versions of this question, and consisted of “definitely not,” “probably not,” “yes, probably,” “yes, definitely,” which were categorized as ‘not intending to complete FOBt screening’ (“definitely not” and “probably not”) and ‘intending to complete FOBt screening’ (“yes, probably” and “yes, definitely”). “Don’t know/not sure” responses were coded as missing.

Self-reported screening uptake among screening-eligible respondents was recorded slightly differently in the four waves of the ABACUS survey, but for the purpose of this study, all responses were dichotomized as ‘never’ (as in never having returned one of the routine colorectal cancer screening test kits sent by the National Health Service BCSP) vs. ‘ever’ screening (i.e. having returned at least one test kit). In *survey* 1, respondents were asked whether they had ever been invited to do a stool test for the National Health Service BCSP. Respondents answering ‘yes’, were asked additional questions about how many times they had received a stool kit and how many times they had taken part. All participants who indicated that they had done at least one test kit were coded as ‘ever’ having participated. In *survey* 2, respondents were asked, ‘Have you ever done the stool test?’ (yes, no, don’t know), with ‘yes’ responses coded as ‘ever’ (1) and ‘no’ responses coded as ‘never’ (0). In *surveys* 3 *and* 4, respondents were asked which of five statements best described them. The statements were based on the stages of the Precaution Adoption Process Model^36^ and were dichotomized to characterize ‘never’ participants as those who were eligible for FOBt screening but reported never having heard of it, never having been invited, or never having done it, and ‘ever’ participants as those who reported having completed the test at least once. Respondents stating ‘don’t know’ or ‘refused’ were coded as missing. A previous study has shown that self-reported ever uptake of screening is highly reliable.^37^

We excluded those with missing data on the cancer worry, intention, and self-reported uptake variables. We report descriptive statistics for all variables for the total sample. We then used logistic regression to examine the relationship between the sociodemographic and cancer worry variables, and FOBt screening intention and uptake. In addition to the unadjusted associations, we compared a series of logistic regression models containing only the sociodemographic variables and ABACUS Wave (Model 1) with four models adding cancer worry frequency (Model 2), cancer worry intensity (Model 3), both (Model 4), or the combined measure of cancer worry (Model 5). We used weighted data for descriptive analyses, as sample weights are highly relevant for prevalence estimation, but relied on unweighted data to test associations, as weights typically do not alter the results of coefficients, and unweighted analysis has been recommended because of being simpler, more transparent, and more accurate (e.g. reduced standard error, reduced risk of overfitting).^38^ As a sensitivity analysis, we also performed the same analyses using the continuous cancer worry variables. For these analyses, we also tested the interaction effect for cancer worry frequency and intensity, and curvilinear effects of cancer worry frequency and intensity on screening intentions and uptake. Stata 12 SE was used for all analyses and alpha levels of 0.05 or less indicated statistical significance.

## Results

The weighted sample characteristics for each wave and for the combined sample are reported in Table 1 (the unweighted sample characteristics are reported in Table S1 in the Online Supplement). The mean age of the sample was 65.2 (SD = 3.3), and 47% were men. Similar to the general population estimates for this age group in England,^39^ the majority of respondents were married or living as married (67%) and of White ethnic origin (95%). About a quarter of respondents (24%) came from social grades D and E, 21% were in grade C2, 24% in grade C1, and 31% in grade A/B. Across all four waves, 69% had participated in FOBt screening at least once, and 81% intended to participate when next invited.

**Table 1.** Weighted sample characteristics per wave and for the combined sample (*N* = 2878).

	ABACUS 1		ABACUS 2		ABACUS 3		ABACUS 4		TOTAL
	*N*=1381		*N*=719		*N*=369		*N*=409		*N*=2878
Age (mean and SD)	65.14	(3.20)	65.06	(3.19)	65.58	(3.27)	65.34	(3.47)	65.21	(3.25)
Gender: *n* (%)										
Male	701	(45.7%)	353	(48.7%)	201	(50.1%)	200	(46.2%)	1455	(47.1%)
Female	680	(54.3%)	366	(51.3%)	168	(49.9%)	209	(53.8%)	1423	(52.9%)
Ethnicity: *n* (%)										
White	1316	(96.1%)	681	(94.5%)	346	(94.5%)	385	(93.8%)	2728	(95.2%)
Other	65	(3.9%)	38	(5.5%)	23	(5.5%)	24	(6.2%)	150	(4.8%)
Marital status: *n* (%)										
Married or living as married	901	(69.7%)	442	(64.7%)	236	(66.7%)	254	(64.8%)	1833	(67.4%)
Single	122	(7.4%)	78	(10.2%)	50	(11.7%)	47	(10.8%)	297	(9.1%)
Widowed, separated or divorced	358	(22.9%)	199	(25.1%)	83	(21.6%)	108	(24.4%)	748	(23.5%)
Social grade: *n* (%)										
A/B	347	(30.9%)	153	(31.3%)	85	(31.1%)	84	(31.3%)	669	(31.1%)
C1	301	(26.5%)	142	(21.6%)	81	(23.2%)	91	(21.6%)	615	(24.2%)
C2	255	(21.4%)	152	(23.0%)	60	(16.4%)	43	(17.8%)	510	(20.7%)
D/E	478	(21.2%)	272	(24.1%)	143	(29.3%)	161	(29.3%)	1054	(24.0%)
Cancer worry frequency: *n* (%)										
Never	484	(32.9%)	360	(49.2%)	142	(36.4%)	176	(42.6%)	1162	(38.7%)
Occasionally	382	(28.6%)	237	(34.7%)	150	(42.0%)	145	(36.4%)	914	(32.8%)
Sometimes	257	(19.5%)	80	(10.5%)	57	(16.2%)	63	(15.4%)	457	(16.3%)
Often	66	(5.1%)	12	(1.4%)	8	(2.3%)	11	(2.6%)	97	(3.5%)
Very often	21	(1.5%)	6	(0.8%)	3	(0.9%)	4	(0.8%)	34	(1.2%)
Missing	171	(12.3%)	24	(3.4%)	9	(2.4%)	10	(2.3%)	214	(7.5%)
Cancer worry intensity: *n* (%)										
Not at all	461	(32.3%)	315	(43.7%)	138	(36.1%)	165	(39.7%)	1079	(36.6%)
Slightly	511	(38.4%)	283	(39.9%)	164	(46.4%)	172	(43.3%)	1130	(40.4%)
Quite	244	(18.4%)	75	(10.4%)	38	(9.6%)	51	(12.4%)	408	(14.5%)
Extremely	110	(7.6%)	18	(2.3%)	19	(5.4%)	11	(2.4%)	158	(5.3%)
Missing	55	(3.4%)	28	(3.7%)	10	(2.5%)	10	(2.2%)	103	(3.2%)
Cancer worry (combined measure): *n* (%)								
No worry	323	(21.8%)	267	(37.1%)	103	(26.8%)	130	(31.1%)	823	(27.5%)
Moderate worry	545	(41.2%)	322	(45.3%)	196	(55.1%)	203	(50.8%)	1266	(45.2%)
High worry	328	(23.8%)	93	(12.7%)	57	(15.0%)	65	(15.7%)	543	(18.9%)
Missing	185	(13.2%)	37	(4.9%)	13	(3.2%)	11	(2.4%)	246	(8.5%)
Uptake: *n* (%)
No	214	(14.7%)	122	(16.1%)	106	(27.0%)	112	(26.5%)	554	(18.3%)
Yes	913	(68.2%)	480	(68.3%)	252	(70.5%)	273	(68.1%)	1918	(68.5%)
Missing	254	(17.1%)	117	(16.6%)	11	(2.6%)	24	(5.4%)	406	(13.2%)
Screening intentions: *n* (%)				
No	180	(12.3%)	105	(14.1%)	58	(12.9%)	48	(10.3%)	391	(12.6%)
Yes	1076	(79.7%)	555	(79.3%)	298	(82.6%)	334	(83.8%)	2263	(80.5%)
Missing	125	(8.0%)	59	(6.6%)	20	(4.5%)	27	(5.9%)	231	(6.9%)

In terms of frequency of worry about cancer, more than a third (39%) never worried about getting cancer, nearly half of the sample (49%) worried occasionally or sometimes, and a minority (5%) worried often or very often (Table 1). In terms of cancer worry intensity, just over a third (37%) did not feel anxious at all when thinking about cancer, 40% were slightly anxious, and 20% were quite or extremely anxious. When these two measures were combined, 28% of the sample was classified as having “no cancer worry,” 45% as having “moderate cancer worry,” and 20% as having “high cancer worry.”

After excluding those with missing data on the cancer worry and intention variables, a sample of *N* = 2463 remained for analysis (Table 2). Of these, 86% intended to take the stool test when they were next invited. FOBt screening intentions were significantly lower in those who were single (79%) or those who were widowed, separated, or divorced (83%), than in those who were married or living as married (88%). There was also a significant social gradient in screening intentions, with fewer of those from lower social grades intending to do the FOBt (C1: 87%, C2: 85%, DE: 81%) compared with those from the highest social grade (AB: 92%), and these associations remained significant when all other sociodemographic variables were adjusted for in Model 1 (Table 2). Model 1 explained 4.2% of variance in the data.

**Table 2.** Unadjusted and adjusted logistic regression for FOBt screening intentions with 95% confidence intervals (*N* = 2463).

	Total	Unadjusted		Model 1		Model 2		Model 3		Model 4		Model 5	
	(%)	OR	CI	OR	CI	OR	CI	OR	CI	OR	CI	OR	CI
Overall	85.8												
Age		0.986	0.952–1.021	0.992	0.957–1.028	0.995	0.960–1.032	0.993	0.958–1.030	0.994	0.958–1.031	0.994	0.959–1.031
Gender													
Male	87.0	Ref		Ref		Ref		Ref		Ref		Ref	
Female	84.5	0.814	0.649–1.021	0.855	0.676–1.081	0.811	0.640–1.028	0.803	0.633–1.019	0.797	0.628–1.011	0.812	0.641–1.030
Ethnicity													
White	86.0	Ref		Ref		Ref		Ref		Ref		Ref	
Non-white	80.2	0.658	0.407–1.064	0.671	0.411–1.094	0.702	0.429–1.148	0.701	0.428–1.148	0.707	0.431–1.159	0.693	0.423–1.135
Marital status													
Married/living as married	88.1	Ref		Ref		Ref		Ref		Ref		Ref	
Single	78.8	0.499	0.353–0.704**	0.556	0.391–0.792**	0.551	0.387–0.785**	0.552	0.387–0.787**	0.550	0.386–0.785**	0.551	0.387–0.785**
Wid/sep/div	82.5	0.636	0.494–0.820**	0.749	0.575–0.975*	0.760	0.583–0.990*	0.759	0.582–0.991*	0.761	0.583–0.993*	0.760	0.583–0.991*
Social grade													
A/B	92.4	Ref		Ref		Ref		Ref		Ref		Ref	
C1	86.8	0.544	0.367–0.805**	0.572	0.385–0.850**	0.587	0.395–0.873**	0.584	0.392–0.869**	0.587	0.394–0.873**	0.583	0.392–0.867**
C2	84.9	0.463	0.312–0.687**	0.476	0.320–0.708**	0.496	0.333–0.738**	0.478	0.321–0.712**	0.483	0.324–0.721**	0.483	0.324–0.719**
D/E	81.0	0.352	0.248–0.499**	0.401	0.280–0.573**	0.418	0.292–0.598**	0.412	0.287–0.590**	0.416	0.290–0.597**	0.411	0.287–0.588**
ABACUS wave													
Wave 1	85.8	Ref		Ref		Ref		Ref		Ref		Ref	
Wave 2	84.8	0.925	0.703–1.218	0.962	0.728–1.271	1.008	0.761–1.335	1.004	0.757–1.331	1.012	0.762–1.343	1.013	0.764–1.343
Wave 3	85.4	0.973	0.690–1.372	1.014	0.715–1.439	0.999	0.703–1.421	1.010	0.710–1.438	1.004	0.705–1.430	1.008	0.709–1.434
Wave 4	87.6	1.172	0.827–1.662	1.254	0.881–1.785	1.275	0.894–1.818	1.291	0.903–1.845	1.291	0.903–1.845	1.286	0.901–1.836
Cancer worry frequency								
Never	82.3	Ref				Ref				Ref			
Occasionally/sometimes	88.4	1.641	1.300–2.072**			1.568	1.234–1.992**			1.151	0.860–1.541		
Often/very often	86.8	1.408	0.813–2.439			1.481	0.847–2.590			1.135	0.608–2.121		
Cancer worry intensity													
Not at all	81.0	Ref						Ref		Ref			
Slightly	89.7	2.033	1.570–2.633**					1.993	1.532–2.593**	1.841	1.350–2.510**		
Quite/extremely	86.6	1.510	1.112–2.051**					1.611	1.175–2.208**	1.482	1.020–2.154*		
Cancer worry (combined)													
None	81.3	Ref										Ref	
Moderate	88.2	1.717	1.332–2.212**									1.665	1.285–2.158**
High	86.6	1.478	1.081–2.020*									1.556	1.127–2.150**
*R*^2^ (Nagelkerke)					0.042		0.052		0.062		0.062	0.053	

wid: widowed; sep: separated; div: divorced.

* *p* < 0.05; ** *p* < 0.01.

We then compared Model 1 with the four models that contained the sociodemographic variables as well as cancer worry frequency (Model 2), cancer worry intensity (Model 3), both (Model 4), or the combined cancer worry measure (Model 5; see Table 2). All models explained significantly more variance than Model 1 (Model 2 $\chi_{\text{diff}}^{2}$(2) = 14.0, *p* < 0.001, Model 3 $\chi_{\text{diff}}^{2}$(2)=28.3, *p* < 0.001, Model 4 $\chi_{\text{diff}}^{2}$(2) = 29.2, *p* < 0.001, and Model 5 $\chi_{\text{diff}}^{2}$(2)=15.8, *p* < 0.001).

Compared with never worrying, worrying about cancer occasionally or sometimes was significantly and positively associated with FOBt screening intentions when all sociodemographic variables were adjusted for (82% vs. 88% reported positive intention; OR = 1.57, 95% CI 1.23–1.99; Model 2), but there was no association with worrying often or very often. Similarly, compared with not being anxious (81% reported positive intention), reporting slight vs. quite a bit or extreme cancer worry intensity increased the odds of intending to do the FOBt (90%; OR = 1.99, 95% CI 1.53–2.59; 87%; OR = 1.61, 95% CI 1.18–2.21, respectively; Model 3). When both measures were combined in a single model (Model 4), cancer worry frequency was no longer significantly associated with FOBt screening intentions, but cancer worry intensity remained significantly associated. For the combined measure (Model 5), both moderate and high levels of cancer worry were significantly and positively associated with FOBt screening intentions, but this model explained less variance (Nagelkerke’s *R*^2^ = .053) than Models 3 or 4 (Nagelkerke’s *R*^2^ = .062 and .062, respectively). The difference between Models 3 and 4 was non-significant (χ^2^_diff_(2) = 0.90, *p* = 0.64). Thus, Model 3 (cancer worry intensity) appeared the most parsimonious.

After excluding those with missing data on the cancer worry and uptake variables, a sample of *N* = 2318 remained for analysis (Table 3). Overall, 78% of respondents reported having done the stool test at least once. Cancer screening uptake was significantly and positively associated with age. Self-reported past FOBt uptake was significantly lower in those who were single (63%) compared with those who were married or living as married (80%), and in those from lower social grades (C2: 78%, DE: 72%) compared with those from the highest social grade (AB: 83%). These associations remained significant when all other sociodemographic variables were adjusted for in Model 1 (Table 3). Model 1 explained 5.8% of the variance in the data.

**Table 3.** Unadjusted and adjusted logistic regression for self-reported FOBt uptake with 95% confidence intervals (*N* = 2318).

	Total	Unadjusted		Model 1		Model 2		Model 3		Model 4		Model 5	
	(%)	OR	CI	OR	CI	OR	CI	OR	CI	OR	CI	OR	CI
Overall	77.5												
Age		1.049	1.018–1.082**	1.059	1.026–1.092**	1.059	1.027–1.093**	1.060	1.027–1.094**	1.058	1.026–1.092**	1.060	1.028–1.094**
Gender													
Male	76.9	Ref		Ref		Ref		Ref		Ref		Ref	
Female	78.2	1.078	0.887–1.310	1.108	0.904–1.358	1.091	0.889–1.339	1.060	0.863–1.303	1.068	0.869–1.312	1.071	0.872–1.315
Ethnicity													
White	77.8	Ref		Ref		Ref		Ref		Ref		Ref	
Non-white	69.4	0.645	0.415–1.003	0.721	0.459–1.135	0.726	0.461–1.143	0.741	0.470–1.169	0.732	0.463–1.157	0.733	0.465–1.155
Marital status													
Married/living as married	80.1	Ref		Ref		Ref		Ref		Ref		Ref	
Single	62.7	0.417	0.309–0.562**	0.483	0.355–0.656**	0.484	0.356–0.659**	0.484	0.356–0.659**	0.485	0.357–0.661**	0.484	0.356–0.658**
Wid/sep/div	76.5	0.810	0.645–1.016	0.867	0.682–1.101	0.871	0.685–1.107	0.879	0.691–1.117	0.874	0.687–1.112	0.877	0.690–1.115
Social grade													
A/B	83.3	Ref		Ref		Ref		Ref		Ref		Ref	
C1	80.2	0.815	0.598–1.111	0.828	0.604–1.136	0.832	0.607–1.141	0.846	0.616–1.161	0.839	0.611–1.153	0.841	0.613–1.154
C2	77.5	0.691	0.504–0.947*	0.696	0.506–0.959*	0.699	0.507–0.964*	0.701	0.508–0.966*	0.690	0.500–0.952*	0.702	0.509–0.967*
D/E	71.7	0.508	0.388–0.665**	0.540	0.408–0.716**	0.542	0.408–0.719**	0.551	0.415–0.732**	0.540	0.406–0.718**	0.545	0.411–0.723**
ABACUS wave													
Wave 1	80.7	Ref		Ref		Ref		Ref		Ref		Ref	
Wave 2	80.2	0.972	0.751–1.257	1.034	0.796–1.343	1.057	0.813–1.375	1.079	0.829–1.406	1.072	0.823–1.397	1.080	0.829–1.407
Wave 3	70.7	0.580	0.439–0.765**	0.610	0.459–0.810**	0.616	0.463–0.819**	0.616	0.463–0.821**	0.624	0.468–0.831**	0.618	0.464–0.823**
Wave 4	71.2	0.593	0.452–0.778**	0.626	0.475–0.825**	0.634	0.480–0.835**	0.642	0.486–0.848**	0.643	0.487–0.850**	0.642	0.486–0.848**
Cancer worry frequency				
Never	76.1	Ref				Ref				Ref			
Occasionally or sometimes	78.1	1.122	0.919–1.370			1.093	0.889–1.344			0.797	0.617–1.031		
Often or very often	83.0	1.540	0.920–2.577			1.485	0.878–2.513			1.013	0.566–1.814		
Cancer worry intensity				
Not at all	72.6	Ref						Ref		Ref			
Slightly	80.5	1.558	1.254–1.935**					1.515	1.213–1.892**	1.722	1.317–2.251**		
Quite and extremely	80.6	1.563	1.188–2.056**					1.545	1.163–2.053**	1.702	1.216–2.384**		
Cancer worry (combined)				
None	73.7	Ref										Ref	
Moderate	78.6	1.314	1.055–1.636*									1.291	1.030–1.618*
High	80.5	1.476	1.114–1.955**									1.457	1.088–1.952*
*R*^2^ (Nagelkerke)					0.058		0.059		0.068		0.070	0.063	

wid: widowed; sep: separated; div: divorced.

*p < 0.05; **p < 0.01.

We then compared Model 1 with the other four models. Model 3 ($\chi_{\text{diff}}^{2}$(2)=16.5, *p* < 0.001), Model 4 ($\chi_{\text{diff}}^{2}$(2)=20.0, *p* < 0.001), and Model 5 ($\chi_{\text{diff}}^{2}$(2)=7.79, *p*=0.02) explained significantly more variance than Model 1, but Model 2 did not ($\chi_{\text{diff}}^{2}$(2)=2.57, *p*=0.28), i.e. cancer worry frequency was not associated with self-reported past uptake of FOBt (Table 3). In Model 3, compared with not being anxious at all (73% uptake), being slightly, or quite a bit or extremely anxious about cancer was associated with increased odds of FOBt uptake (81%; OR = 1.52, 95% CI 1.21–1.89; and 81%; OR = 1.55, 95% CI 1.16–2.05, respectively). Cancer worry frequency was not significantly associated with FOBt uptake in Model 4 when cancer worry intensity was also included in the model. For the combined cancer worry measure, moderate and high levels of worry were significantly associated with FOBt screening uptake (Model 5), but this model explained less variance than Models 3 and 4, which again explained most variance (Nagelkerke’s *R*^2^=.068 and .070, respectively). There was no difference between these models in the amount of variance explained ($\chi_{\text{diff}}^{2}$(2)=3.49, *p*=0.18). Thus, Model 3 (cancer worry intensity) again appeared the most parsimonious.

We repeated all analyses using the continuous cancer worry variables and an interaction term for cancer worry frequency and intensity to test their additive effects. We also tested for curvilinear effects in these analyses. The full results are reported in the Online Supplement. Briefly, both cancer worry frequency and intensity were significantly associated with FOBt screening intentions, both linearly and curvilinearly (Online Supplement Table S2). When both were combined into a single model, either separately or their interaction, only cancer worry intensity remained significantly associated with screening intentions, while the variance explained increased only marginally compared with a model with only cancer worry intensity (*R*^2^=.066 vs. *R*^2^=.065). The results were very similar for self-reported FOBt screening uptake (Table 2, and Online Supplement Table S3). The results from these sensitivity analyses therefore also suggest that the most parsimonious model of FOBt screening intentions and uptake is one with cancer worry intensity.

## Discussion

This study found that being at least slightly and at least occasionally worried about cancer was relatively common, with nearly two out of three people being either moderately or very worried on the measure that combined frequency and intensity of cancer worry. A comparison of the two measures indicated that respondents did not necessarily worry about cancer often, but that many experienced moderate to high levels of worry when they thought about it. Furthermore, it was this level of cancer worry intensity, rather than the frequency of worrying, that seemed to be associated with CRC screening intention and uptake. Our findings therefore seem to suggest that, contrary to common practice, a single item on cancer worry intensity rather than frequency could be used when trying to explain variance in uptake of colorectal cancer screening.

This is the first study of FOBt screening in the UK to include measures of both cancer worry frequency and intensity. It is also the first study to explicitly examine which of these measures is more strongly associated with intentions and with (self-reported) screening uptake.

It should be noted that previous research did not just find differences between different aspects of cancer worry, but also important inconsistencies according to screening modality (FOBt vs. endoscopic screening), programme (cervical vs. breast vs. colorectal), population (US vs. UK vs. Singapore), and whether measures of screening intentions or uptake are used. For this reason, it is difficult to make strong claims about the practical implications of the findings. Future research should investigate psychological antecedents of cancer worry intensity. While simply increasing worry about cancer would be potentially unethical and an inappropriate way to increase engagement with cancer screening, it is possible that what underlies our observation relates to more general perceptions of cancer, or indeed cancer screening (e.g. lack of perceived risk and optimistic bias), which could be addressed without necessarily ‘scaring’ people into attending cancer screening.

The sociodemographic patterns in FOBt screening intentions and uptake in our study were similar to previous studies,^6,7^ with intentions and uptake lower in those from more deprived backgrounds and those who are single. Screening intentions and uptake were also lower in those from non-White ethnic backgrounds, although these differences were not significant in our study. Our study may have been underpowered to detect these differences due to the small percentage of those from a non-White ethnic background in our sample (5%), which is nevertheless consistent with the ethnic distribution for this age group.^39^ As would be expected, we found a small but statistically significant positive association between age and ‘ever’ having done the FOB test, as those who are older would have received more invitations to complete the test, but we did not find an association between age and intentions to do the test when next invited. Self-reported ‘ever’ uptake of the FOBt (69%) was similar to self-reported ever uptake in other studies in the UK (70%),^37,40^ and has been shown to be highly consistent with actual uptake of FOBt screening.^37^

This study had some limitations. It is the first study to suggest that cancer worry intensity, rather than frequency, may be more strongly associated with uptake of screening for colorectal cancer, although these constructs were measured using single items that have inherent validity problems, and face validity was the only accessible psychometric property of the measures used in the current study. More work is needed to validate measures of cancer worry frequency and intensity, and future studies should try to replicate our findings to see if measures of cancer worry in large population-based studies should be changed from frequency to intensity items. We note that the single item measure relating to cancer worry in the US HINTS has already been changed from a frequency to an intensity-based item from the 2003, 2005, and 2008 to the 2011–2013 and 2017 surveys.^33^ The questions about cancer worry frequency and cancer worry intensity were about cancer in general, not specifically about colorectal cancer. This may have affected the strength of the associations, as previous research shows that attitudes may differ between cancer types,^5,41^ and future studies should use measures specific to colorectal cancer. In addition, few people scored the highest two levels of cancer worry frequency (often and very often). We therefore chose to collapse these categories, but the small sample size, even in the collapsed category, may have limited the power to detect any effects of frequent cancer worry on screening intention and uptake, and future studies may wish to oversample these levels of cancer worry frequency to formally test these associations. A further limitation is that this study only measured intensity and frequency of cancer worry, while various literature reviews of cancer worry in the general population suggest that there may be a variety of sources of cancer worry that may have different behavioural effects.^8,25,26^ In our recent review of the qualitative literature, we provide a comprehensive taxonomy of cancer worries related to cancer screening, including cancer worry as a general and diffuse worry, and worries about specific aspects of cancer, such as treatment, death, or the social consequences of a diagnosis.^25^ A recent population-based survey of self-reported cancer screening uptake found differential behavioural associations by type of cancer worry,^30^ and future studies should therefore include more comprehensive measures of cancer worry whenever possible. Items used to assess general feelings of cancer worry, such as those used in the current study, may nevertheless give a meaningful general overview of whether the cancer worry emotion tends to favour uptake of a particular type of screening or not. Furthermore, excluding people with missing data introduced a certain amount of selection bias, as indicated by the level of intention and uptake in the final analytic sample (86% and 77% respectively). We adjusted for the effect of sociodemographic confounders, but there may be other confounding variables, such as family history of colorectal cancer and physician recommendation, that may influence the association between cancer worry and screening uptake, and could be included in future studies. Finally, due to the cross-sectional nature of the study, it is not possible to draw any conclusions about the causal nature of the relationships or their temporal development. Prospective, longitudinal studies could examine how cancer worry frequency and intensity develop over time and in response to screening attendance.

## Conclusion

This large cross-sectional analysis was the first to compare cancer worry frequency versus cancer worry intensity in the context of CRC screening. Our findings suggest that cancer worry intensity, rather than frequency, is informative when understanding engagement with CRC screening using gFOBt. Future studies should use prospective designs and objective uptake across different CRC screening modalities, and other cancer screening programmes.

## Supplemental Material

Supplemental material for Cancer worry frequency vs. intensity and self-reported colorectal cancer screening uptake: A population-based study Click here for additional data file. Supplemental Material for Cancer worry frequency vs. intensity and self-reported colorectal cancer screening uptake: A population-based study by Charlotte Vrinten, Sandro Stoffel, Rachael H Dodd, Jo Waller, Yoryos Lyratzopoulos and Christian von Wagner in Journal of Medical Screening
