# Supplementary material for: Cancer worry frequency vs. intensity and self-reported colorectal cancer screening uptake: A population-based study
Source: J Med Screen. 2019 May 1;26(4):169–78. doi: 10.1177/0969141319842331 (PMC6854611; doi:10.1177/0969141319842331)
Supplement: Supplemental material for Cancer worry frequency vs. intensity and self-reported colorectal cancer screening uptake: A population-based study [file Supplemental_Material.pdf]

Online supplement to "Cancer worry frequency vs intensity and self-reported colorectal cancer screening uptake: a population-based study" by C Vrinten, S Stoffel, RH Dodd, J Waller, Y Lyratzopoulos, C von Wagner.

Table S1. Descriptive statistics for overall sample without weights (age 60-70 years without any cancer diagnosis)

	ABACUS 1		ABACUS 2		ABACUS 3		ABACUS 4		TOTAL	
	N=1,381		N=719		N=369		N=409		N=2,878	
Age (mean and SD)	65.14	(3.20)	65.06	(3.19)	65.58	(3.27)	65.34	(3.47)	65.21	(3.25)
**Gender:** n (%)										
Male	701	(50.8%)	353	(49.1%)	201	(54.5%)	200	(48.9%)	1,455	(50.6%)
Female	680	(49.2%)	366	(50.9%)	168	(45.5%)	209	(51.1%)	1,423	(49.4%)
**Ethnicity:** n (%)										
White	1316	(95.3%)	681	(94.7%)	346	(93.8%)	385	(94.1%)	2,728	(94.8%)
Other	65	(4.7%)	38	(5.3%)	23	(6.2%)	24	(5.9%)	150	(5.2%)
**Marital status:** n (%)										
Married or living with as married	901	(65.2%)	442	(61.5%)	236	(64.0%)	254	(62.1%)	1,833	(63.7%)
Single	122	(8.8%)	78	(10.8%)	50	(13.6%)	47	(11.5%)	297	(10.3%)
Widowed, separated or divorced	358	(25.9%)	199	(27.7%)	83	(22.5%)	108	(26.4%)	748	(26.0%)
**Social grade:** n (%)										
A/B	347	(25.1%)	153	(21.3%)	85	(23.0%)	84	(22.2%)	669	(23.5%)
C1	301	(21.8%)	142	(19.7%)	81	(22.0%)	91	(24.0%)	615	(21.6%)
C2	255	(18.5%)	152	(21.1%)	60	(16.3%)	43	(11.3%)	510	(17.9%)
D/E	478	(34.6%)	272	(37.8%)	143	(38.8%)	161	(42.5%)	1,054	(37.0%)
**Cancer worry (frequency):** n (%)										
Never	484	(35.0%)	360	(50.1%)	142	(38.5%)	176	(43.0%)	1,162	(40.4%)
Occasionally	382	(27.7%)	237	(33.0%)	150	(40.7%)	145	(35.5%)	914	(31.8%)
Sometimes	257	(18.6%)	80	(11.1%)	57	(15.4%)	63	(15.4%)	457	(15.9%)
Often	66	(4.8%)	12	(1.7%)	8	(2.2%)	11	(2.7%)	97	(3.4%)
Very often	21	(1.5%)	6	(0.8%)	3	(0.8%)	4	(1.0%)	34	(1.2%)
Missing	171	(12.4%)	24	(3.3%)	9	(2.4%)	10	(2.4%)	214	(7.4%)
**Cancer worry (intensity):** n (%)										
Not at all	461	(33.4%)	315	(43.8%)	138	(37.4%)	165	(40.3%)	1,079	(37.5%)
Slightly	511	(37.0%)	283	(39.4%)	164	(44.4%)	172	(42.1%)	1,130	(39.3%)
Quite	244	(17.7%)	75	(10.4%)	38	(10.3%)	51	(12.5%)	408	(14.2%)
Extremely	110	(8.0%)	18	(2.5%)	19	(5.1%)	11	(2.7%)	158	(5.5%)
Missing	55	(4.0%)	28	(3.9%)	10	(2.7%)	10	(2.4%)	103	(3.6%)
**Cancer worry (combined)**: n (%)										
No fear	323	(23.4%)	267	(37.1%)	103	(27.9%)	130	(31.8%)	823	(28.6%)
Moderate fear	545	(39.5%)	322	(44.8%)	196	(53.1%)	203	(49.6%)	1,266	(44.0%)
High fear	328	(23.8%)	93	(12.9%)	57	(15.4%)	65	(15.9%)	543	(18.9%)
Missing	185	(13.4%)	37	(5.1%)	13	(3.5%)	11	(2.7%)	246	(8.5%)
**Uptake:** n (%)										
No	214	(15.5%)	122	(17.0%)	106	(28.7%)	112	(27.4%)	554	(19.2%)
Yes	913	(66.1%)	480	(66.8%)	252	(68.3%)	273	(66.7%)	1,918	(66.6%)
Missing	254	(18.4%)	117	(16.3%)	11	(3.0%)	24	(5.9%)	406	(14.1%)
**Screening intentions:** n (%)										
No	180	(13.0%)	105	(14.6%)	58	(15.4%)	48	(11.7%)	391	(13.6%)
Yes	1076	(77.9%)	555	(77.2%)	298	(79.3%)	334	(81.7%)	2,263	(78.4%)
Missing	125	(9.1%)	59	(8.2%)	20	(5.3%)	27	(6.6%)	231	(8.0%)

Table S2. Adjusted logistic regression for FOBt screening intentions with 95% confidence intervals (N=2,463)

	Cancer worry frequency				Cancer worry intensity			
	Model 1 - Linear		Model 2 - Curvilinear		Model 3 - Linear		Model 4 - Curvilinear	
	OR	CI	OR	CI	OR	CI	OR	CI
**Age**	0.994	0.959 - 1.031	0.996	0.961 - 1.033	0.993	0.958 - 1.029	0.993	0.958 - 1.030
**Gender**								
Male	Ref		Ref		Ref		Ref	
Female	0.819	0.646 - 1.037	0.811	0.640 - 1.028	0.820	0.647 - 1.038	0.805	0.635 - 1.022
**Ethnicity**								
White	Ref		Ref		Ref		Ref	
Non-white	0.682	0.417 - 1.114	0.701	0.428 - 1.147	0.675	0.413 - 1.104	0.706	0.431 - 1.158
**Marital status**								
Married/living as married	Ref		Ref		Ref		Ref	
Single	0.551	0.387 - 0.785**	0.549	0.385 - 0.782**	0.558	0.392 - 0.795**	0.543	0.381 - 0.775**
Wid/sep/div$	0.758	0.582 - 0.988*	0.762	0.585 - 0.994*	0.756	0.580 - 0.986*	0.754	0.578 - 0.984*
**Social grade**								
A/B	Ref		Ref		Ref		Ref	
C1	0.580	0.390 - 0.862**	0.586	0.394 - 0.871**	0.577	0.388 - 0.858**	0.588	0.395 - 0.875**
C2	0.487	0.327 - 0.725**	0.495	0.333 - 0.738**	0.476	0.320 - 0.708**	0.482	0.324 - 0.719**
D/E	0.407	0.284 - 0.582**	0.417	0.291 - 0.597**	0.395	0.276 - 0.565**	0.418	0.291 - 0.598**
**ABACUS wave**								
Wave 1	Ref		Ref		Ref		Ref	
Wave 2	1.015	0.767 - 1.344	1.015	0.766 - 1.345	1.018	0.769 - 1.348	0.993	0.749 - 1.318
Wave 3	1.032	0.727 - 1.466	1.007	0.709 - 1.432	1.045	0.735 - 1.484	1.011	0.711 - 1.439
Wave 4	1.288	0.904 - 1.836	1.280	0.898 - 1.826	1.314	0.921 - 1.874	1.272	0.889 - 1.819
**Cancer worry frequency**								
Linear (1-5)	1.216	1.064 - 1.391**	2.080	1.261 - 3.431**				
Curvilinear (1-25)			0.889	0.801 - 0.986*				
**Cancer worry intensity**								
Linear (1-4)					1.248	1.084 - 1.438**	5.248	2.859 - 9.635**
Curvilinear (1-16)							0.721	0.631 - 0.823**
R^2 (Nagelkerke)	0.048		0.051		0.049		0.065	

$wid = widowed, sep = separated, div=divorced   * p<0.05; ** p<0.01

Table S2. Adjusted logistic regression for FOBt screening intentions with 95% confidence intervals (N=2,463; cont'd.)

	Cancer worry frequency and intensity				Cancer worry frequency*intensity (interaction)			
	Model 5 - Linear		Model 6 - Curvilinear		Model 7 - Linear		Model 8 - Curvilinear	
	OR	CI	OR	CI	OR	CI	OR	CI
**Age**	0.994	0.959 - 1.030	0.994	0.958 - 1.031	0.995	0.960 - 1.032	0.994	0.958 - 1.031
**Gender**								
Male	Ref		Ref		Ref		Ref	
Female	0.810	0.639 - 1.027	0.799	0.629 - 1.014	0.808	0.637 - 1.024	0.796	0.627 - 1.011
**Ethnicity**								
White	Ref		Ref		Ref		Ref	
Non-white	0.680	0.416 - 1.112	0.710	0.433 - 1.164	0.689	0.421 - 1.127	0.714	0.436 - 1.171
**Marital status**								
Married/living as married	Ref		Ref		Ref		Ref	
Single	0.554	0.389 - 0.790**	0.541	0.379 - 0.773**	0.548	0.384 - 0.781**	0.542	0.380 - 0.775**
Wid/sep/div$	0.759	0.583 - 0.990*	0.757	0.580 - 0.988*	0.760	0.583 - 0.992*	0.758	0.581 - 0.989*
**Social grade**								
A/B	Ref		Ref		Ref		Ref	
C1	0.581	0.390 - 0.863**	0.590	0.396 - 0.878**	0.585	0.393 - 0.869**	0.589	0.396 - 0.877**
C2	0.482	0.324 - 0.717**	0.487	0.327 - 0.726**	0.485	0.326 - 0.723**	0.487	0.326 - 0.726**
D/E	0.400	0.280 - 0.573**	0.421	0.294 - 0.604**	0.406	0.283 - 0.581**	0.423	0.295 - 0.606**
**ABACUS wave**								
Wave 1	Ref		Ref		Ref		Ref	
Wave 2	1.033	0.779 - 1.369	1.003	0.755 - 1.333	1.039	0.783 - 1.377	0.999	0.751 - 1.327
Wave 3	1.046	0.736 - 1.487	1.010	0.709 - 1.438	1.036	0.729 - 1.472	1.008	0.707 - 1.436
Wave 4	1.316	0.922 - 1.878	1.274	0.890 - 1.822	1.314	0.920 - 1.876	1.269	0.887 - 1.816
**Cancer worry frequency**								
Linear (1-5)	1.121	0.955 - 1.317	1.170	0.663 - 2.067	1.553	1.098-2.196*	0.915	0.333-2.515
Curvilinear (1-25)			0.981	0.874 - 1.101			0.978	0.817-1.170
**Cancer worry intensity**								
Linear (1-4)	1.166	0.985 - 1.380	4.685	2.368 - 9.267**	1.510	1.124-2.030*	3.909	1.543-9.903**
Curvilinear (1-16)			0.732	0.633 - 0.847**			0.725	0.610-0.860**
**Cancer worry frequency*intensity**								
Linear (1-20)					0.867	0.761 – 0.988**	1.182	0.806 - 1.734
Curvilinear (1-400)							0.996	0.982 – 1.011
R^2 (Nagelkerke)	0.050		0.065		0.053		0.066	

$wid = widowed, sep = separated, div=divorced   * p<0.05

R^2 changes for FOBt screening intention models

- Model 1 vs Model 2 (cancer worry frequency): $p = 0.0317$
- Model 3 vs Model 4 (cancer worry intensity): $p < 0.0001$
- Model 5 vs Model 6 (cancer worry frequency and intensity): $p < 0.0001$
- Model 7 vs Model 8 (cancer worry frequency*intensity): $p = 0.0004$

- Model 5 vs Model 7 (no interaction vs interaction): $p = 0.0363$

Table S3. Adjusted logistic regression regression for self-reported FOBt uptake with 95% confidence intervals (N=2,318)

	Cancer worry frequency				Cancer worry intensity			
	Model 1 - Linear		Model 2 - Curvilinear		Model 3 - Linear		Model 4 - Curvilinear	
	OR	CI	OR	CI	OR	CI	OR	CI
**Age**	1.059	1.027 - 1.093**	1.059	1.026 - 1.093**	1.060	1.027 - 1.094**	1.060	1.027 - 1.094**
**Gender**								
Male	Ref		Ref		Ref		Ref	
Female	1.095	0.892 - 1.344	1.096	0.892 - 1.345	1.065	0.867 - 1.308	1.064	0.866 - 1.307
**Ethnicity**								
White	Ref		Ref		Ref		Ref	
Non-white	0.723	0.460 - 1.137	0.720	0.458 - 1.134	0.728	0.462 - 1.149	0.744	0.472 - 1.174
**Marital status**								
Married/living as married	Ref		Ref		Ref		Ref	
Single	0.483	0.355 - 0.657**	0.483	0.355 - 0.657**	0.486	0.357 - 0.661**	0.480	0.352 - 0.654**
Wid/sep/div$	0.871	0.685 - 1.106	0.870	0.684 - 1.106	0.876	0.689 - 1.114	0.877	0.689 - 1.115
**Social grade**								
A/B	Ref		Ref		Ref		Ref	
C1	0.832	0.607 - 1.140	0.830	0.606 - 1.139	0.838	0.611 - 1.150	0.849	0.618 - 1.165
C2	0.700	0.508 - 0.964*	0.698	0.506 - 0.961*	0.696	0.505 - 0.959*	0.704	0.510 - 0.970*
D/E	0.543	0.409 - 0.719**	0.540	0.407 - 0.717**	0.537	0.405 - 0.713**	0.556	0.419 - 0.738**
**ABACUS wave**								
Wave 1	Ref		Ref		Ref		Ref	
Wave 2	1.051	0.808 - 1.367	1.052	0.808 - 1.369	1.089	0.837 - 1.417	1.071	0.822 - 1.396
Wave 3	0.614	0.462 - 0.816**	0.616	0.463 - 0.820**	0.627	0.471 - 0.833**	0.615	0.462 - 0.818**
Wave 4	0.631	0.479 - 0.832**	0.632	0.479 - 0.833**	0.651	0.493 - 0.859**	0.636	0.481 - 0.840**
**Cancer worry frequency**								
Linear (1-5)	1.054	0.942 - 1.180	0.967	0.617 - 1.517				
Curvilinear (1-25)			1.019	0.926 - 1.121				
**Cancer worry intensity**								
Linear (1-4)					1.211	1.070 - 1.369**	2.664	1.563 - 4.543**
Curvilinear (1-16)							0.833	0.740 - 0.939**
R^2 (Nagelkerke)	0.058		0.058		0.064		0.069	

$wid = widowed, sep = separated, div=divorced   * p<0.05; ** p<0.01

Table S3. Adjusted logistic regression regression for self-reported FOBt uptake with 95% confidence intervals (N=2,318; cont'd.)

	Cancer worry frequency and intensity				Cancer worry frequency*intensity (interaction)			
	Model 5 - Linear		Model 6 - Curvilinear		Model 7 - Linear		Model 8 - Curvilinear	
	OR	CI	OR	CI	OR	CI	OR	CI
**Age**	1.059	1.026 - 1.093**	1.057	1.024 - 1.091**	1.059	1.027 - 1.093**	1.057	1.024 - 1.091**
**Gender**								
Male	Ref		Ref		Ref		Ref	
Female	1.073	0.873 - 1.318	1.077	0.875 - 1.324	1.073	0.873 - 1.319	1.077	0.875 - 1.324
**Ethnicity**								
White	Ref		Ref		Ref		Ref	
Non-white	0.728	0.462 - 1.149	0.736	0.465 - 1.163	0.730	0.463 - 1.151	0.736	0.466 - 1.164
**Marital status**								
Married/living as married	Ref		Ref		Ref		Ref	
Single	0.487	0.357 - 0.662**	0.480	0.352 - 0.654**	0.485	0.356 - 0.660**	0.481	0.353 - 0.655**
Wid/sep/div$	0.873	0.687 - 1.110	0.868	0.682 - 1.105	0.873	0.687 - 1.110	0.868	0.682 - 1.106
**Social grade**								
A/B	Ref		Ref		Ref		Ref	
C1	0.836	0.609 - 1.147	0.842	0.613 - 1.157	0.839	0.611 - 1.151	0.842	0.613 - 1.157
C2	0.691	0.501 - 0.953*	0.690	0.500 - 0.953*	0.694	0.503 - 0.957*	0.690	0.500 - 0.953*
D/E	0.533	0.402 - 0.707**	0.543	0.408 - 0.721**	0.537	0.405 - 0.713**	0.543	0.408 - 0.722**
**ABACUS wave**								
Wave 1	Ref		Ref		Ref		Ref	
Wave 2	1.078	0.827 - 1.404	1.055	0.809 - 1.376	1.078	0.827 - 1.404	1.054	0.808 - 1.375
Wave 3	0.625	0.470 - 0.831**	0.622	0.467 - 0.828**	0.622	0.467 - 0.827**	0.621	0.466 - 0.827**
Wave 4	0.650	0.492 - 0.858**	0.635	0.480 - 0.840**	0.648	0.491 - 0.855**	0.634	0.480 - 0.839**
**Cancer worry frequency**								
Linear (1-5)	0.925	0.805 - 1.063	0.557	0.333 - 0.931*	1.049	0.785-1.401	0.606	0.251 -1.467
Curvilinear (1-25)			1.109	0.997 - 1.233			1.094	0.933 - 1.282
**Cancer worry intensity**								
Linear (1-4)	1.273	1.092 - 1.483**	3.759	2.053 - 6.882**	1.423	1.083-1.871*	4.039	1.715 - 9.513**
Curvilinear (1-16)			0.784	0.688 - 0.893**			0.776	0.659 - 0.914**
**Cancer worry frequency*intensity**								
Linear (1-20)					0.944	0.842 - 1.059	0.968	0.677 - 1.385
Curvilinear (1-400)							1.002	0.988 - 1.015
R^2 (Nagelkerke)	0.064		0.073		0.065		0.073	

$wid = widowed, sep = separated, div=divorced   * p<0.05

R^2 changed for self-reported FOBt screening uptake models

- Model 1 vs Model 2 (cancer worry frequency): p= 0.6977
- Model 3 vs Model 4 (cancer worry intensity): p= 0.0030
- Model 5 vs Model 6 (cancer worry frequency and intensity): p= 0.0012
- Model 7 vs Model 8 (cancer worry frequency*intensity): p= 0.0058

- Model 5 vs Model 7 (no interaction vs interaction): p= 0.3327
